# The influence of microenvironment on tumor immunotherapy

**DOI:** 10.1111/febs.15028

**Published:** 2019-08-22

**Authors:** Jieying Zhang, Zhaopeng Shi, Xiang Xu, Zuoren Yu, Jun Mi

**Affiliations:** ^1^ Department of Biochemistry and Molecular Cell Biology Key Laboratory of Cell Differentiation and Apoptosis of Chinese Ministry of Education Shanghai Jiao Tong University School of Medicine China; ^2^ Research Center for Translational Medicine East Hospital TongJi University School of Medicine Shanghai China; ^3^ Hongqiao International Institute of Medicine Tongren Hospital Shanghai Jiao Tong University School of Medicine China

**Keywords:** cancer‐associated fibroblasts, tumor immunotherapy, tumor microenvironment

## Abstract

Tumor immunotherapy has achieved remarkable efficacy, with immune‐checkpoint inhibitors as especially promising candidates for cancer therapy. However, some issues caused by immunotherapy have raised attention, such as limited efficacy for some patients, narrow antineoplastic spectrum, and adverse reactions, suggesting that using regulators of tumor immune response may prove to be more complicated than anticipated. Current evidence indicates that different factors collectively constituting the unique tumor microenvironment promote immune tolerance, and these include the expression of co‐inhibitory molecules, the secretion of lactate, and competition for nutrients between tumor cells and immune cells. Furthermore, cancer‐associated fibroblasts, the main cellular components of solid tumors, promote immunosuppression through inhibition of T cell function and extracellular matrix remodeling. Here, we summarize the research advances in tumor immunotherapy and the latest insights into the influence of microenvironment on tumor immunotherapy.

## Graphical abstract

The tumor microenvironment plays an important role in tumor immunotherapy. Different factors collectively constituting the unique tumor microenvironment promote immune tolerance, including the expression of co‐inhibitory molecules, the secretion of lactate, and competition for nutrients between tumor cells and immune cells. Furthermore, cancer‐associated fibroblasts, the main cellular components of solid tumors, promote immunosuppression through inhibition of T cell function and extracellular matrix remodeling.

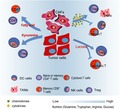

**Abbreviations**
A2AR: adenosine A2a receptor
AHR: aryl hydrocarbon receptor
ARG1: arginase 1
CAF: cancer‐associated fibroblast
CAR‐T: chimeric antigen receptor T cell therapy
CD73: cluster of differentiation 73
Chi3L1: chitinase‐3‐like‐1
COX‐2: cyclooxygenase‐2
CSF1: colony stimulating factor 1
CTLA‐4: cytotoxic T lymphocyte antigen‐4
CTL: cytotoxic T lymphocyte
DC: dendritic cell
ECM: extracellular matrix
EGCG: epigallocatechin‐3‐gallate
FAP: fibroblast activation protein
FASL: Fas ligand
FSP1: fibroblast specific 1
FOXP3: forkhead box P3
GITR: glucocorticoid‐induced tumor necrosis factor receptor
HCC: hepatocellular carcinoma
HIF‐1α: hypoxia‐induced factor 1α
IDO: Indoleamine 2,3-Dioxygenase
IFN: interferon
IL: interleukin
LAG-3: lymphocyte Activating 3
MDSC: myeloid‐derived suppressor cell
MHC: major histocompatibility complex
MSI: microsatellite instability
MSS: microsatellite stability
NHL: non‐Hodgkin lymphoma
NK: natural killer
NO: nitric oxide
PD‐1: programmed death 1
PGE2: prostaglandin E2
STAT: signal transducer and activator of transcription
TAM: tumor‐associated macrophage
Teff: effector T
TGF‐β: transforming growth factor β
TIM-3: T Cell Immunoglobulin Mucin 3
TIGIT: T Cell Immunoreceptor With Ig And ITIM Domains
TME: tumor microenvironment
TNF: tumor necrosis factor
Treg: regulatory T
VEGF: vascular endothelial growth factor

## Introduction

The increasing incidence and mortality of cancer is a major threat to human health, and finding safe and effective therapies is a big challenge to researchers throughout the world. Surgical excision, radiotherapy, and chemotherapy are traditional oncology treatments, but do not provide uncontroversial benefits for patient survival. Although gene‐targeted therapies prolong tumor patients’ survival, relapse after treatment is still a big challenge to oncologists. With the rapid development of oncology, immunology, molecular biology, and other related disciplines, immunotherapy has brought revolutionary changes in cancer treatment. Immunotherapy aims at activating the patient's immune system to kill cancer cells, and includes chimeric antigen receptor T‐cell therapy (CAR‐T), immune‐checkpoint blockade, and tumor vaccines.

In 1989, the use of the CAR‐T cells was first approved to significantly enhance antitumor efficiency independent of the major histocompatibility complex (MHC) 1. Chimeric antigen receptors are expressed on T cells and specifically target tumor surface antigens and kill tumor cells 2, 3, 4. They consist of a tumor‐specific antigen, tumor‐associated antigen binding domain, a hinge domain, a transmembrane domain, and an intracellular signaling domain. For example, the US Food and Drug Administration‐approved CAR‐T drugs tisagenlecleucel (CTL019) and axicabtagene ciloleucel (KTE‐C10) have been used to treat recurrent or refractory patients with all and certain types of non‐Hodgkin lymphoma (NHL) 5.

Immune checkpoints regulate the immune response and play important roles in the self‐tolerance of the immune system. Inhibitory checkpoint pathways are potential therapeutic targets of cancer. Immune‐checkpoint blockade blocks harmful co‐inhibitory molecules and activates the patient's immune system to enhance the function of antitumor T lymphocytes 6. Cytotoxic T lymphocyte antigen‐4 (CTLA‐4) binds its ligand, B7, to produce inhibitory signals, inhibit T cell activation, and protect tumor cells from T cell attack. In the meantime, programmed death 1 (PD‐1) binds its ligand, PD‐L1 and PD‐L2, to inhibit the signaling pathway that activates T cells. PD‐L1 is expressed on tumor cells, immune cells, and epithelial cells while PD‐L2 is induced exclusively on antigen‐presenting cells 7, 8. The blocking antibodies against CTLA‐4 (ipilimumab) and PD1 (nivolumab and pembrolizumab) have shown exciting efficacy in cancer therapy 9, 10, 11, 12. Moreover, an increasing number of therapeutic antibodies against PD1/PD‐L1 or the co‐inhibitory receptors lymphocyte Activating 3 (LAG‐3), T Cell Immunoglobulin Mucin 3 (TIM‐3), T Cell Immunoreceptor With Ig And ITIM Domains (TIGIT), and CD73 are under clinical trial 8, 13.

Tumor vaccines were the first tested approach to killing tumor cells by activating antigen‐specific immune responses. The tumor vaccines sipuleucel‐T, MEGA‐A3, and nelipepimut‐S 14, 15, 16 are induced by tumor‐specific antigen or tumor‐associated antigen from prostate cancer, lung cancer, and breast cancer, respectively. However, tumor vaccines alone have never shown clear benefit to cancer patients, probably due to tumor heterogeneity and plasticity. Therefore, we will not discuss the factors affecting the efficacy of tumor vaccines in this review.

However, some issues that emerged from immunotherapy, such as limited efficacy for some patients, narrow antineoplastic spectrum, and adverse reactions, indicating regulators of tumor immune response, are more complicated than first thought. The tumor microenvironment (TME) is the cellular niche consisting of tumor cells, fibroblasts, immune cells, signaling molecules, and the extracellular matrix (ECM). Numerous studies have demonstrated that the TME plays essential roles in tumor development, progression, and tumor recurrence post‐therapy. Recent studies indicate that the TME regulates the efficacy of tumor response to immune therapy. A better understanding of the influence of the TME on the immune response will contribute to improving the efficacy of immunotherapy. Tumor cells and cancer‐associated fibroblasts (CAFs) are the primary and secondary cell populations in tumors, and the activity of immune cells inside a tumor directly determines the antitumor response; therefore, we will focus on the effects of these type of cells on immunotherapy.

## Regulation of tumor immune response by tumor cells

Cytokines, chemokines, and metabolites derived from tumor cells have a significant impact on TME, such as transforming growth factor β (TGF‐β), interleukin (IL)‐10, and CCXL15. Tumor cells inhibit the function of natural killer (NK) cells, CD8^+^ T cells, and cytotoxic T lymphocytes (CTLs), which helps tumor cells escape from recognition and attack by the immune system. Most tumor cells express a high level of stem cell factor, which induces migration of mast cells to the tumor site by interacting with c‐kit. Moreover, mast cells inactivate T cells and NK cells and inhibit their antitumor activity by expressing pro‐inflammatory factors. Colony stimulating factor 1 (CSF1) produced by tumor cells contributes to the transformation and differentiation of tumor‐associated macrophages (TAMs) and decreased granulocyte‐specific chemokines in CAFs. Treatment with a CSF1 receptor inhibitor and a CXCR2 antagonist suppressed recruitment of polymorphonuclear myeloid‐derived suppressor cells (MDSCs) to tumors and showed strong antitumor effects 17.

Tumors also modify certain inflammatory cell types to render them tumor promoting rather than tumor suppressive; in particular, the immune cells in cancer‐associated chronic inflammation promote tumor progression 18, 19. For example, high mobility group box 1 is released from tumor cells and binds to Toll‐like receptor 4 and receptor (its receptor) for advanced glycation end product ligands, further activating regulatory T (Treg) cells and releasing cytokines such as IL‐10, promoting immune escape and the proliferation of tumor cells 20.

Hypoxia is a common feature of solid tumors due to the fast growth of tumor cells. Hypoxia in the tumor microniche helps shield cancer cells from immune attack and inhibits immune killing functions 21, 22, 23. Hypoxia‐induced factor 1α (HIF‐1α) is a critical regulator of adaptive responses to hypoxia and regulates glucose metabolism, angiogenesis, cell proliferation, invasion, and metastasis 24. HIF‐1α increases the expression of PD‐L1 in tumor cells and inhibits the response of T cells.

Hypoxia also engages in an important crosstalk that has serious implications for immunological recognition of tumors. Tumor cells decrease the expression of MHC or tumor antigens to avoid being recognized and cleared by immune cells 25. MHC and tumor antigen expression are critical for the migration and maturation of dendritic cells (DCs) and tumor‐specific T cells 26. Tumors with a high density of infiltrating T cells are called ‘hot tumors’. The PD‐L1 on tumor cells shuts down the T cell immune response to protect tumor cells by targeting PD‐1 on the activated T cells 27, 28.

The adenosine A2a receptor (A2AR) is a member of the G protein‐coupled receptor superfamily, and adenosine is the preferred endogenous agonist. A2AR plays an important role in many biological functions, such as cardiac rhythm and circulation, cerebral and renal blood flow, immune function, pain regulation, and sleep. A2AR suppresses activation of immune cells. The expression of A2AR was correlated with the protein level of HIF‐1α, CD8, forkhead box P3 (FOXP3), and CD73 in the head and neck squamous cell carcinoma. Inhibition of A2AR decreased the infiltrating CD4^+^ FOXP3^+^ Treg cells and markedly enhanced the antitumor response of CD8^+^ T cells by attenuating hypoxia‐HIF‐1α signaling; the combined treatment with CTLA‐4/PD1 enhanced the antitumor effects 29, 30.

Compared with normal cells, tumor cells require a large amount of energy and synthesis of biomass material for survival and growth. In order to match this requirement, tumor cells reprogram their catabolic and anabolic metabolism 31, 32. The Warburg effect is a well‐known metabolic feature in which tumor cells exhibit an increased dependence on the glycolytic pathway for ATP generation and biosynthesis, even under aerobic conditions. Meanwhile, cancer cells also have increased ingestion of fatty acids and amino acids, for example glutamine, tryptophan, and arginine. We will elaborate the dysfunctions of immune cells caused by the deficiency of these amino acids in immune cells in the following section.

## Regulation of tumor immune response by cancer‐associated fibroblasts

CAFs are the major components of the tumor stroma, characterized by high expression of fibroblast activation protein α, platelet‐derived growth factor receptor β and prolyl‐4‐hydroxylase. CAFs are transformed in the epithelial–mesenchymal transition, endothelial‐mesenchymal transition or progenitor/stem cell differentiation, but mainly activated from adjacent quiescent fibroblast activation.

**Figure 1:**
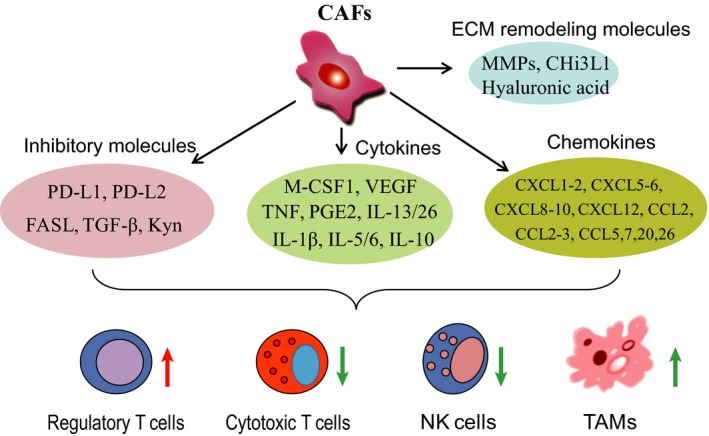
The cytokines/chemokines and enzymes secreted by cancer‐associated fibroblasts. The cancer‐associated fibroblast‐secreted or exported cytokines, chemokines, inhibitory and ECM remodeling molecules together regulate immune cell function. Inhibitory molecules mainly include PD‐L1, PD‐L2, FASL, TGF‐β and kynurenine (Kyn). Cytokines and chemokines include M‐CSF1, VEGF, TNF, PGE2, IL‐13/26, IL‐1β, IL‐5/6, IL‐10, CXCL1‐2, CXCL5‐6, CXCL8‐10, CXCL12, CCL2, CCL2‐3, CCL5, etc. Representative ECM remodeling molecules are matrix metalloproteinase (MMPs), Chi3L1, and hyaluronic acid. Together, these molecules secreted by CAFs suppress NK cell and cytotoxic T cell activity.

CAFs are prone to robust glycolysis and secrete a large amount of cytokines and chemokines, as showed in Fig. 1 33. CAFs promote tumor immunosuppression by secreting abundant cytokines and chemokines, such as CXCL12, CXCL8, IL‐6, tumor necrosis factor (TNF), TGF‐β, CCL2, vascular endothelial growth factor (VEGF) and co‐regulatory molecules B7H1/B7DC 34, 35. CAF‐derived CCL17 and CXCL12 attract Treg cells migrating to cutaneous basal cell carcinoma. Targeting CXCL12 or CXCL12 receptor CXCR4 has a synergistic effect with anti‐PD‐L1 treatment; the combined treatment caused T cell accumulation and cancer regression 36. CAFs also suppressed NK cell activity by inhibiting the expression of the activating receptors, such as NKp44, NKp30, DNAM‐1, and poliovirus receptor (PVR/CD155) 37, 38.

Meanwhile, CAFs increased the expression of Fas and PD‐1 on the T cell surface, resulting in CD8^+^ T cells being exhausted in an antigen‐dependent manner via PD‐L2 and Fas ligand (FASL) 35, 39. High levels of CAF‐secreted IL‐6 induced tumor immunosuppression by recruiting MDSCs and upregulating PD‐L1 expression, impairing the efficacy of anti‐PD‐L1 immunotherapy against hepatocellular carcinoma (HCC), and IL‐6 blockade increased the efficacy of PD‐L1 treatment 40. Interestingly, tumor‐associated stromal cells/CAFs themselves also expressed PD‐L1 to suppress CD8^+^ antitumor immune responses in response to the inflammatory secretome from human colon cancer cells, and pre‐inflammatory cytokine TNFα 41 and IL‐1β induced CXCL8/CCL5 secretion in the co‐culture of CAFs and triple negative breast cancers 42, suggesting that the inflammation also regulates the immunological response of CAFs/stromal cells in the TME. In addition, CAFs in HCC regulate the survival and activation of neutrophils through the IL‐6–signal transducer and activator of transcription (STAT) 3–PD‐L1 signaling pathway and consequently affect the function of T cells 43. Even after high‐dose irradiation, CAFs maintain their powerful immunosuppressive effect over activated T cells 44.

In addition, CAFs protect tumor cells from CTL attack by remodeling the ECM 45, 46. There are strong correlations between the chemoattractant IL‐16 and the density of CD3^+^, CD45RO^+^, and CD8^+^ cells. IL‐6 regulates the cytokine network, which determines leucocyte density and phenotype in the TME 47, 48, 49. CAFs also produce hyaluronic acid to recruit TAMs to the TME, and the depletion of hyaluronic acid synthase significantly decreased the density of TAMs in the TME 50. Glycoprotein chitinase‐3‐like‐1 (Chi3L1) is highly expressed in CAFs and involved in fibrotic disorders. Depletion of Chi3L1 in CAFs promoted tumor growth and increased the infiltration of CD4^+^ T cells and CD8^+^ T cells in tumors 51.

Mesenchymal stem cells are an important source of CAFs, especially during tumor initiation. The mesenchymal stem cells in cancer have many biomarkers and functions in common with CAFs 52, 53. Although the role of inhibitor of nuclear factor κB kinase β is controversial in tumorigenesis, nuclear factor‐κB activation‐induced IL‐6 in mesenchymal cells promotes tumorigenesis of skin, pancreatic, intestinal, and mammary cancer 54, 55, 56. In a pre‐leukemic disorder mouse model, mesenchymal cells induced genotoxic stress including oxidative stress and DNA damage response through inflammatory signaling in hematopoietic stem and progenitor cells 57.

In brief, CAF‐derived cytokines/chemokines regulate tumor immune evasion and promote tumor growth and metastasis 58, and targeting CAF therapy could promote a tumor immune response. Fibroblast activation protein (FAP) is a major marker of CAFs. The SynCon FAP DNA vaccine induced both CD8^+^ and CD4^+^ T cell activation and inhibited tumor growth and metastasis through reducing FAP^+^ CAFs 59, 60. Besides DNA vaccines, targeting FAP has synergistic effects with immune‐checkpoint blockades. Ferritin is the core of a photosensitizer carrier. A FAP‐specific single chain variable fragment‐conjugated ferritin nanoparticle‐based photoimmunotherapy suppressed CXCL12 secretion and ECM deposition, and significantly enhanced T cell infiltration 61. However, systematical depletion of CAFs by targeting FAP triggers recognition of multipotent bone marrow stromal cells and cachexia 62.

## Regulation of tumor immune response by immune cells

During tumor progression, tumor‐infiltrating immune cells such as Treg cells, TAMs and MDSCs secrete inflammatory cytokines to promote angiogenesis and tumor cell proliferation, invasion, and metastasis.

### T lymphocytes

Regulatory T cells are a subset of CD4^+^ T cells with immunosuppression that influence tumor immunotherapy and vaccine activation. Treg cells are characterized by the expression of CD4, CD25, and FOXP3, although they may show phenotypic diversity and functional heterogeneity in different types of tumor and tissues. Hypoxia enhanced Treg abundance *in vitro* and *in vivo* by upregulation of FOXP3 24. Removing Treg cells in the tumor microniche or dampening the function of Treg cells is a strategic approach to tumor therapy.

Moreover, hypoxia markedly enhances expression of CD137 on activated T lymphocytes, which is an important molecular target to augment antitumor immunity 22. Ablation of HIF‐1α in CD11c^+^ cells resulted in a higher frequency of short‐lived effector cells, enhanced CD8^+^ T cell expansion, and increased IL‐12 expression by splenic DCs 63. Downregulation of HIF‐1α enhanced NK‐mediated antitumor immunity, especially when synergized with B7‐1‐mediated immunotherapy 64.

The US Food and Drug Administration recently approved the CD25 blocker daclizumab, which promotes the secretion of interferon (IFN) ‐γ by selectively reducing the number of Treg cells 65. In a clinical trial for breast cancer therapy, the combination of daclizumab and tumor vaccines successfully removed Treg cells and increased the number of CD4^+^ and CD8^+^ effector T (Teff) cells in the meantime 66. Glucocorticoid‐induced tumor necrosis factor receptor (GITR) and OX40 (CD134) are members of the TNF receptor family that are expressed in CD4^+^CD25^+^ Treg cells. The ligand or the agonist antibody of GITR or OX40 inhibits the immunosuppressive function of CD4^+^CD25^+^ Treg cells and promotes cytokine secretion. The combination of GITR and OX40 agonist is promising for inhibition of Treg‐mediated suppression 67.

### Tumor‐associated macrophages

Based on function and cytokine secretion, macrophages are divided into two subgroups, classical activation (M1), and alternative activation (M2). Much evidence shows that macrophages play critical roles in tumor cell clearance. Several immunosuppressive signals impair their function, particularly in solid tumors 68. Macrophages in solid tumors are called TAMs, and are similar to M2 macrophage TAMs; they are a subpopulation of tumor‐infiltrating immune cells and contribute to tumor progression and metastasis 69.

Tumor‐associated macrophages release a number of cytokines, chemokines, and enzymes that suppress the effector function of CD4^+^ and CD8^+^ T cells. TAMs as well as tumor cells recruited Treg cells to the tumor site by secreting CCL20 or CCL22 and fostered immune privilege in colorectal cancer or ovarian carcinoma, respectively 70. Conditional TAM ablation blocked Treg‐cell recruitment and inhibited tumor growth by decreasing the level of CCL20 in xenografted mice 71. TAMs originated from circulating CCR2^+^ monocytes degraded the ECM and remodeled the TME 72. And targeting the CCL2/CCR2 chemokine axis reduced TAM accumulation at the metastatic site, restored antitumor T cell function, and disrupted the immunosuppressive TME 73. In addition, arginase 1 (ARG1) is upregulated by TAMs and tumor cells, and its expression inhibits T cell activation by reducing arginine entry into tumor‐infiltrating immune cells 74.

Moreover, TAMs suppress immune cell function by expressing multiple receptors or ligands of the inhibitory receptors (PD‐L1, PD‐L2, B7‐1). B7‐H4 is a tumor‐associated transmembrane protein that is upregulated on the surface of cancer cells and TAMs. Inhibition of B7‐H4 in NHL cells promoted T cell immunity and cytotoxic activity of NHL‐reactive T cells 75. The expression of B7‐H4 decreased CD4^+^ T cell responses via binding to semaphorin 3a 76. TAMs induce the expression of PD‐L1 by the secretion of IFN‐γ through the Janus kinase–STAT3 and phosphoinositide 3‐kinase–AKT signaling pathways. PD‐1's ligands, PD‐L1 and PD‐L2, cause T cell exhaustion and promote tumor immune escape.

The lactate produced by HIF‐1α‐dependent anaerobic metabolism also affects the polarization of TAMs 77, 78. Meanwhile, hypoxia‐induced secretion of B7 homologue 3 promoted phorbol 12‐myristate 13‐acetate‐induced THP‐1 cell transformation into the M2‐type of TAMs. Hypoxia also increased the expression of ARG1, VEGF and macrophage‐derived chemokine CCL22 by activating mitogen‐activated protein kinase signaling in TAMs 74, 79. Glycocalyx‐mimicking nanoparticles were used to reverse TAMs, which increased immunostimulatory IL‐12 secretion and reduced the level of immunosuppressive IL‐10, ARG1, and CCL22. The reversion of TAMs improved the effect of anti‐PD‐L1 cancer immunotherapy by suppressing STAT6 and/or activating nuclear factor‐κB 80, 81, suggesting blockade of B7‐H4, PD1, or PD‐L1 as an approach for tumor therapy.

### Myeloid‐derived suppressor cells

MDSCs are a heterogeneous population of immature myeloid cells that have a remarkable ability to suppress T cell responses. They comprise immature granulocytes, monocytes, and DCs. They prevent the T cell‐mediated adaptive immune response and the NK‐ or TAM‐mediated innate immune system from killing tumor cells 82.

MDSCs inhibit Teff cell function in many ways. They inhibit the function of CD8^+^ T cells by producing nitric oxide (NO) through highly expressing nitric oxide synthase 2. MDSCs also induce the formation of Treg cells by secreting IL‐10 and TGF‐β 83. Moreover, MDSCs affect T cell functions by consuming nutrients in the TME that are essential to T cell activity. For example, an arginine defect leads to Teff cell inactivation 84. In addition, MDSCs promote TAM differentiation and promote tumor proliferation by dimerization of CD45 85. In an xenograft mouse model, MDSCs inhibited the formation and cytotoxicity of NK cells by decreasing the expression of natural killer group 2 member D (NKG2D) and IFN‐γ secretion 86.

## Nutritional competition in tumor microniche

**Figure 2:**
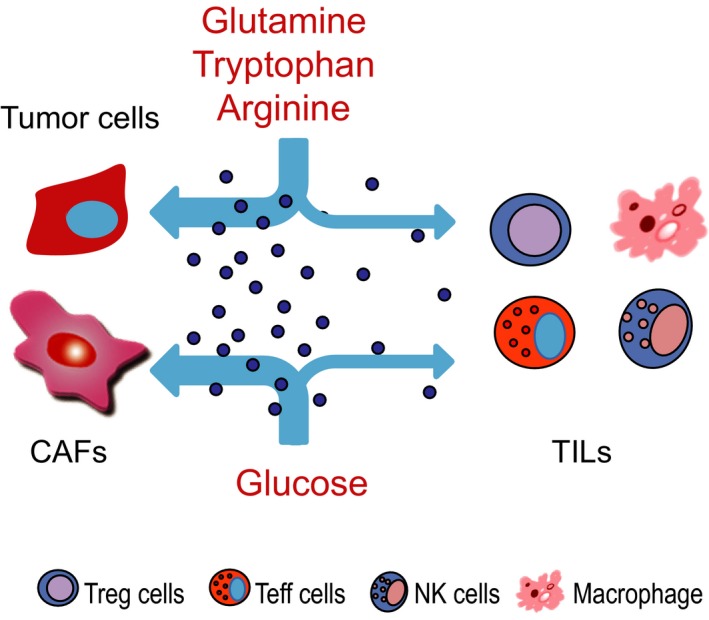
The nutritional competition between tumor cells and immune cells inside tumors. The competition‐caused deficiency of glucose and a couple of amino acids are known to affect the function of immune cells, including Treg, Teff, NK cells and macrophages. TILs, Tumor infiltrating lymphocytes.

The amount of glucose ingested by tumor cells is 10 times more than that of normal cells. Nutrient competition between cancer cells and T cells influences tumor progression. Tumor‐imposed nutrient restrictions leads to T cell hyporesponsiveness (Fig. 2). The fuels glucose and amino acids contribute to the metabolic functions of tumor cells, including tumor growth and metastasis, immune tolerance, and chemoresistance 87.

Glucose deficiency leads to a reduction of glycolytic metabolites, impairing production of IFN‐γ and function of cytotoxic T cells 88, 89. For example, glucose deprivation increased TGF‐β secretion and decreased phosphoenolpyruvate production in activated CD4^+^ T cells. The deficiency of phosphoenolpyruvate led to the suppression of the Ca^2+^/nuclear factor of activated T cells signal and effector functions by increasing Ca^2+^ reuptake. Phosphoenolpyruvate carboxykinase 1 prolonged the survival of xenografted mice by increasing phosphoenolpyruvate concentration 90.

Glutamine is essential for lipid synthesis and is required for maintaining the TCA cycle as well as being an immunomodulator. The l‐glutamine transporter ASCT2 (also known as SLC1a5) is a vital regulator of T cell receptor‐stimulated glutamine uptake and metabolic activities in naïve CD4^+^ T cells. ASCT2 is upregulated in several types of cancer 91. ASCT2 deficiency impaired the differentiation of naïve CD4^+^ T cells and production of Th1 and Th17 cells 92. Other amino acid transporters, such as l‐leucine transporter (LAT1) and γ‐aminobutyric acid transporter 1, are crucial for naïve T cells homeostasis, activation, and differentiation 93.

Tryptophan is one of the essential amino acids; its metabolite kynurenine and related metabolic enzymes are implicated in innate and adaptive immune tolerance 94. Indoleamine 2,3‐dioxygenase and tryptophan 2,3‐dioxygenase 2 (TDO2) are crucial rate‐limiting enzymes that catalyze conversion of tryptophan to produce kynurenine 95, 96, 97. Fibroblasts, macrophages, endothelial, and tumor cells express IDO, while TDO2 expression is limited in the liver.

Tryptophan metabolites/enzymes suppress inflammation by recruiting Treg cells and inhibiting Teff cell proliferation 98, 99. Hepatic CAF‐derived IL‐6 induces tumor immune escape by IDO upregulation in DCs 100. CAFs suppress killing activity of NK cells through downregulation of PVR/CD155, a ligand of activating NK receptor, and the IDO inhibitor 1‐methyl tryptophan inhibits this suppression 38. (−)‐Epigallocatechin‐3‐gallate inhibits IDO expression by blocking IFN‐γ‐induced Janus kinase–protein kinase C‐δ–STAT1 signaling, suggesting that IDO is a potential target for immunotherapy 101.

Arginine is another amino acid affecting the antitumor activity of T cells 102. Arginine is rapidly catabolized by MDSCs and macrophages, resulting in arginine deficiency in the TME. Arginine deficiency leads to the protein biosynthesis‐mediated cellular exhaustion of T cells, which causes T cells to lose their antitumor activity 103, 104, 105. Arginine activates a gene expression program that enhances the bioenergetic profile of T cells, leading to a central memory‐like T cell state and improved antitumor activity 102, 106. Immunotherapies targeting arginine metabolism are also promising strategies.

## Accumulation of abnormal metabolites in the tumor environment

### Lactic acid

**Figure 3:**
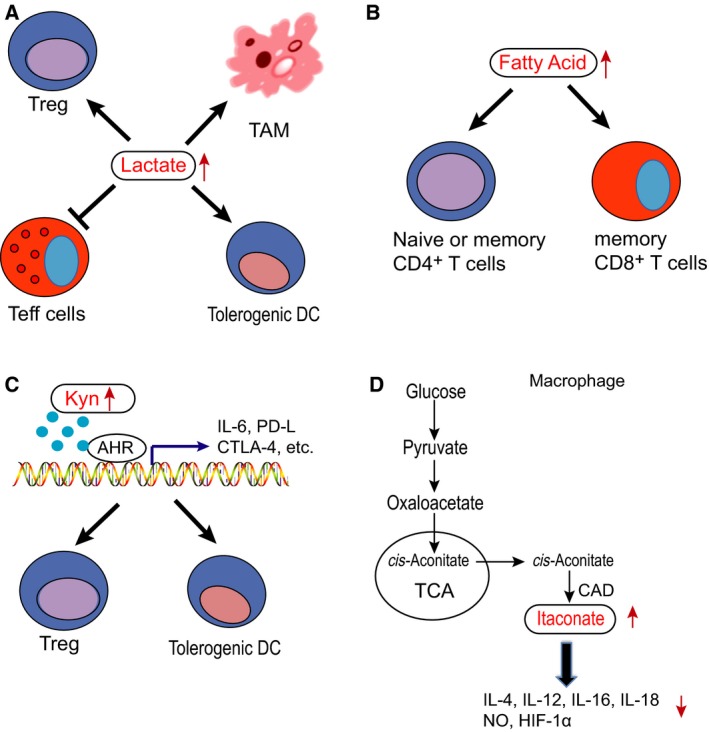
The regulation of metabolites in immune cells. The metabolites are known regulators of immune cell function, such as lactate, fatty acid, kynurenine and itaconate. (A) High level of extracellular lactate inhibits the function of human Teff cells, and promotes the function of Treg cells, TAMs and tolerogenic DCs. (B) Naïve or memory CD4^+^ T cells and memory CD8^+^ T cells are prone to utilize fatty acids as energy sources. (C) Kynurenine binding to AHR promotes the functions of DCs and Treg cells, contributing to immune evasion. (D) Itaconate regulates macrophage inflammatory responses by altering cytokine production. Itaconate suppressed the production of IL‐1β, IL‐18, IL‐6, IL‐12, NO, and HIF‐1α.

Tumor cells produce a large amount of lactic acid, CO_2_, and other metabolites through glycolysis. As showed in Fig. 3A, a high level of extracellular lactate inhibits the proliferation and cytokine production of human CTLs 107. Excessive lactic acid led to an acidic environment, which reduced the concentration of arginine in the TME by inducing ARG1 expression in macrophages, and consequently suppressed the activation, proliferation, and functions of human CD8^+^ T cells 108, 109. Treatment with the acidic buffer bicarbonate inhibited tumor growth by increasing T cell infiltration, and improved antitumor responses of combined therapy with anti‐CTLA‐4, anti‐PD1, or adoptive T cell transfer 110.

Furthermore, intracellular lactic acid as a glycolysis product suppresses T cell glycolysis by inhibiting the phosphoinositide 3‐kinase–AKT–mechanistic target of rapamycin pathway 111. Blocking of the lactate transporter monocarboxylate transporter 1 caused lactic acid accumulation, resulting in a decrease in glycolytic flux in T cells 112.

### Fatty acids

Fatty acids are critical components of macromolecules and cell membrane. Memory T cells are prone to utilize fatty acids as energy sources (Fig. 3B). Fatty acid metabolism influences immune response through regulating lipogenesis. For examples, the survival and differentiation of CD8^+^ T cells was coupled with fatty acid metabolic reprogramming. Acetyl‐CoA carboxylase 1 (ACC1), an enzyme converting acetyl‐CoA to malonyl‐CoA, is a carbon donor for long‐chain fatty acid synthesis. T cell‐specific deletion of ACC1 impaired peripheral persistence and homeostatic proliferation of CD8^+^ T cells in naïve mice and caused a severe defect in Ag‐specific CD8^+^ T cell accumulation 113.

Most tumors are accompanied by inflammation. In turn, inflammation promotes tumor progression by activating the cyclooxygenase‐2 (COX‐2)–prostaglandin E2 (PGE2) pathway. COX‐2 is the rate‐limiting enzyme for arachidonic acid metabolism; its catabolite, PGE2, promotes inflammation. COX‐2 overexpression increased IL‐10 and TGF‐β secretion from DCs, and recruited CD4^+^ T regulatory type 1 cells in glioma 114. Inhibition of COX‐2 decreases the production of PGE2 and IL‐10, and restores IL‐12 secretion 115. Therefore, targeting the COX‐2–PGE2 pathway to suppress inflammation may be a promising strategy for cancer prevention and therapy 116.

### Kynurenine

Kynurenine is a key intermediate metabolite of tryptophan that is involved in inflammation and immune modulation 96, 117. Kynurenine and kynurenic acid are regarded as agonists of the endogenous aryl hydrocarbon receptor (AHR) 118, 119. AHR regulates the functions of DCs, macrophages, NK cells, innate lymphoid cells, Th17 cells, Th22 cells, and Treg cells, which are involved in the immune system 95 (Fig. 3C). After binding to corresponding ligands, AHR is translocated into the nucleus from the cytoplasm, and forms a heterdimer with AHR nuclear translocation protein 95. Through binding to their gene promoter region, this heterodimer combined with some co‐transcription factors promotes the transcription of cytokines including IL‐10 in DCs and NK cells 120, 121 and IL‐6 in cancer cells and macrophages 122, 123. Upon activated by kynurenine, AHR increases the proliferation of DCs and Treg cells. Kynurenine also promotes apoptosis of Teff cells especially Th1 cells 124, 125. Furthermore, AHR is found to be essential to induce IDO expression 119.

### Itaconate

Itaconate is one of the highly induced metabolites in lipopolysaccharide‐activated macrophages 126, 127. It is produced by the enzyme immune responsive gene 1 (Irg1), which is highly expressed in activated macrophages during inflammation 128. Itaconate regulates the metabolism and function of macrophages 126, 129. *Irg1* gene silencing significantly decreased endogenous itaconate levels in macrophages, dramatically reducing antimicrobial ability during bacterial infections 128. In lipopolysaccharide‐activated macrophages, itaconate inhibits the expression of succinate dehydrogenase, a vital pro‐inflammatory regulator 129, 130. Itaconate also regulates macrophages’ inflammatory responses by altering cytokine production. Itaconate suppressed the production of IL‐1β, IL‐18, IL‐6, IL‐12, NO, and HIF‐1α, but not TNF‐α 126 (Fig. 3D). Furthermore, itaconate directly alkylated cysteine residues of Kelch‐like ECH‐associated protein 1 (KEAP1), an E3 ubiquitin ligase, which plays an important role in the antioxidant response 129. Under stress, alkylated KEAP1 stabilizes nuclear related factor‐2 (Nrf2) and Nrf2 consequently translocates into the nucleus to upregulate its downstream genes. Nrf2 plays an important role in antioxidation and anti‐inflammation 129, 131, suggesting itaconate may regulate the tumor immune response.

## Prospective

The TME contributes to tumor growth and metastasis. Tumors are heterogeneous, so the responses to immunotherapies are various. However, a recent discovery showed that a subset of TGF‐β‐responsive squamous cell carcinoma initiating cells are refractory to adoptive T cell transfer immunotherapy in a skin cancer model due to expression of CD80, leading to dampening of cytotoxic T cell responses, and upon TGF‐β‐blocking or CD80 ablation, tumor initiating cells become vulnerable 132. In addition, a herpes simplex virus‐derived immunotherapy, talimogene laherparepvec (T‐VEC), resulted in a high response rate and improved the efficacy of anti‐PD‐1 therapy in patients with advanced melanoma by changing the TME 133, 134. These studies suggest that DCs present antigens of virus‐killed tumor cells to tumor‐specific T cells, and TME change successfully increases immune recognition of cancer 26.

**Figure 4:**
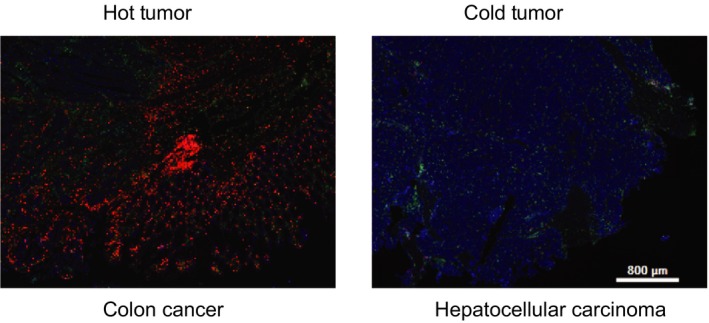
Representative ‘hot’ tumor and ‘cold’ tumor revealed by immunofluorescence staining. Colon cancer and HCC served as a representative hot tumor and cold tumor, respectively. The immunofluorescence staining on cryosections of human colon cancer and HCC was performed with antibodies against FSP1 and CD4. FSP1 (green) is a CAF marker, CD4 (red) represents the CD4^+^ lymphocytes and 4′,6‐diamidino‐2‐phenylindole (blue) stains the nucleus.

Based on the number of infiltrating lymphocytes in them, tumors are simply classified into two subgroups, ‘hot tumors’ *versus* ‘cold tumors’ (Fig. 4). Therefore, immunotherapy targeting tumor‐infiltrating lymphocytes should vary due to the numbers of immune cells inside the different types of tumors. In cold tumors, such as HCC and melanoma, tumor‐infiltrating immune cells are much fewer than in hot tumors. The fewer infiltrating immune cells in the tumors is possibly due to the deficiency of tumor antigen presentation and/or defects of the antigen receptors of the immune cells. Therefore, increasing the tumor‐infiltrating immune cells by CAR‐T technology is a promising strategy to improve the efficacy of tumor immunotherapy.

Inside hot tumors, immune cells are dysfunctional and inactivated due to increased suppressive regulatory immune cells and/or inhibitory receptors. An increasing amount of data have demonstrated that hypoxia, cytokines/chemokines, and metabolites in the TME increase differentiation and proliferation of regulatory immune cells and regulate inhibitory receptor expression. Immune‐checkpoint blockades, such as PD1, PD‐L1, and CTLA‐4 blockades, successfully cured a certain number, but not all of the tumors. The underlying mechanism is poorly understood. Clinical practices have demonstrated that immune‐checkpoint blockades are more effective for colorectal cancers showing microsatellite instability (MSI) than colorectal cancers showing microsatellite stability (MSS), suggesting there are multiple pathways impairing cytotoxic T cell function besides PD1/PD‐L1, and that PD1/PD‐L1 blockades only take effect on cytotoxic T cells expressing PD1. Therefore, it is necessary to detect PD1/PD‐L1 expression prior to PD1/PD‐L1 blockade application.

A recent study showed that elevation of the extracellular potassium concentration impairs T cell function, and lowering intracellular potassium concentration in tumor‐specific T cells by overexpressing the potassium channel Kv1.3 improves effector functions *in vitro* and *in vivo* 135, 136. In addition, the first‐line medicine for type 2 diabetes, metformin, which degrades PD‐L1 protein level and improves the effects of CTLA‐4 blockade, successfully suppressed tumor growth *in vitro* and *in vivo* 137, 138. These findings suggest changing the TME also enhances the efficacy of the immune‐checkpoint blockades.

Furthermore, the cold tumor *versus* the hot tumor is a relative concept. Even in a hot tumor, patients with MSI colon cancer have a higher response rate to the PD1 blockade than the patients with MSS cancer since the MSI colon cancer cells are believed to homing more tumor‐specific T cells than the MSS ones. In general, the TME plays an important role in tumor immunotherapy, no matter whether the tumor is hot or cold. Therefore, carefully dissecting the TME is critical to improving the efficacy of tumor immunotherapy, particularly to these emerging co‐activators such as specific metabolites and/or ions. Combined immunotherapy with targeting of components of the TME could be a promising strategy.

## Materials and methods

### Multi‐color immunohistochemistry

Tumor samples were obtained with the understanding and written consent of patients from the 9th people's hospital affiliated to Shanghai Jiao‐Tong University School of Medicine; the study was approved by the Ethical Review Board of the Medical Faculty of the Shanghai Jiao‐Tong University School of Medicine. Fresh tumors were fixed in 10% formalin for 48 h, and then embedded in paraffin. Tumors were sliced into sections of 5 μm of thickness. The slides were stored at −20 °C before being deparaffinizing in xylene and then rehydrated in a series of concentrations of alcohol (100%, 90% and 70%), successively. Antigen was retrieved in boiled Antigen Repair buffer (Perkin Elmer, San Jose, CA) for 15 min. After a pre‐incubation with blocking buffer at room temperature for 10 min, the sections were incubated at room temperature for 1 h with rabbit anti‐human CD4 (Abcam, Cambridge, MA, clone SP7, 1 : 100). A secondary horseradish peroxidase‐conjugated antibody (PerkinElmer) was added and incubated at room temperature for 10 min. The signal was amplified using diluted Opal520 buffer (1 : 150) at room temperature for 10 min. At the second round, the slides were incubated with the rabbit anti‐human fibroblast specific 1(FSP1) (CST, 1 : 10 000); after antigen retrieval, the signal was amplified in diluted Opal570 buffer. Eventually, the multispectral image was collected with a Leica DM2500 fluorescence microscope (Buffalo Grove, IL).

## Conflict of interest

The authors declare no conflict of interest.

## Author contributions

JZ and ZS performed the experiments and wrote the manuscript; XX drew the illustration graphics; ZY and JM reviewed and the manuscript.
